# Morphological Trait Analysis Showed the Existence of a Migratory Ecotype in the Fall Armyworm, *Spodoptera frugiperda*

**DOI:** 10.3390/insects17010095

**Published:** 2026-01-14

**Authors:** Jiajie Ma, Yishu Sun, Xiaoting Sun, Yifei Song, Wei He, Bo Chu, Xianming Yang, Kongming Wu

**Affiliations:** 1 State Key Laboratory of Cotton Bio-Breeding and Integrated Utilization, School of Life Sciences, Henan University, Kaifeng 475001, China; 15736725579@163.com; 2 State Key Laboratory for Biology of Plant Diseases and Insect Pests, Institute of Plant Protection, Chinese Academy of Agricultural Sciences, Beijing 100193, China; sunyishuwy@163.com (Y.S.); sxiaoting1993@163.com (X.S.); 12216115@zju.edu.cn (Y.S.); zqbxming@163.com (X.Y.); 3 Agricultural Information Institute, Chinese Academy of Agricultural Sciences, Beijing 100081, China; 4 College of Tropical Crops, Hainan University, Haikou 570228, China; 5 Institute of Insect Sciences, College of Agriculture and Biotechnology, Zhejiang University, Hangzhou 310058, China; 6 School of Plant Protection and Environment, Henan Institute of Science and Technology, Xinxiang 453003, China; hewei6006@163.com; 7 College of Plant Protection, Henan Agricultural University, Zhengzhou 450002, China; chubo907@163.com

**Keywords:** fall armyworm, migratory eco-type population, morphological traits, flight ability

## Abstract

*Spodoptera frugiperda* (fall armyworm, FAW) has rapidly spread across Asia and Africa in recent years, with its seasonal long-distance migration ability serving as the biological basis driving its region-wide outbreaks. Although the migratory biology of FAW has been extensively studied, it remains unclear whether there is stable differentiation between migratory and non-migratory individuals. In this study, we revealed the significant differences in morphological parameters between migratory populations and laboratory-reared populations. The migratory populations exhibited a greater body length and width and forewing size, as well as a lower body weight, compared to the laboratory colony. After three generations of indoor rearing, the migrants’ morphology and flight capacity converged to the laboratory phenotype, indicating the existence of a migratory ecotype in FAW. Through further investigation, a method for identifying the migratory ecotype of FAW was proposed based on the corrected wing loading (WL) and forewing aspect ratio (FA), which was successfully applied to distinguish individuals of the migratory ecotype in field populations. Our results confirm that FAWs exhibit stable differentiation into a migratory ecotype, and using WL and FA provides a robust, field-deployable tool for regional FAW monitoring, early warning systems, and targeted FAW control.

## Simple Summary

Determining whether species differentiation exists between migratory and non-migratory ecotypes of *Spodoptera frugiperda* (fall armyworm, FAW) is crucial for elucidating its migratory mechanisms and for developing effective monitoring and early-warning systems. This study systematically compared morphological traits between migratory populations and laboratory-reared strains and analyzed the trends in the morphological traits and flight capability of the migratory populations after three generations of indoor rearing. The results indicated that the migratory population of FAW exhibited more favorable morphological traits for flight compared to the laboratory colony, and these traits decayed rapidly under indoor rearing conditions over three generations. Furthermore, based on model analysis of the morphological parameters of the migratory population, a method for identifying migratory ecotype FAW was established and applied to distinguish these individuals in field populations. These findings confirm that FAWs have adopted a strategy of developing a migratory ecotype to enhance habitat adaptability, providing a theoretical basis for the development of monitoring and forecasting technologies for migratory populations.

## 1. Introduction

Migration is an evolutionarily acquired seasonal adaptation strategy in insects, enabling them to spatially escape unfavorable living conditions and locate new suitable habitats [1]. This behavior occurs over broad geographical scales and involves massive biomass flows, thereby exerting profound impacts on the ecosystems of both the source and destination regions [2,3,4,5]. While a considerable proportion of migratory insects benefit local ecosystems through natural pest suppression and plant pollination [6,7,8], the majority of migratory pests reduce crop yields and facilitate the spread of pathogens [2,4,9,10]. Consequently, long-distance migration of pests exacerbates the agricultural economic and ecological impacts [2,11,12].

Intraspecific variation is prevalent among migratory insects [13]. In certain species, migratory individuals exhibit distinct behavioral, physiological, and morphological traits [14,15,16,17]. Wing polymorphism is relatively common in some taxa; for instance, aphids, brown planthoppers (*Nilaparvata lugens*), and white-backed planthoppers (*Sogatella furcifera*) have developed long-winged morphs specialized for migration [16,18]. Additionally, color morphs within the same species display markedly divergent flight behaviors. Gregarious locusts (*Locusta migratoria*) with dark-brown body coloration exhibit nearly four times longer flight durations and farther flight distances compared to solitary locusts with green body coloration [19]. Likewise, under field conditions, brown-colored individuals of *Oedaleus decorus* exhibit roughly six times higher take-off rates than green morphs [20]. Similarly, melanized forms of the oriental armyworm (*Mythimna separata*) show significantly reduced flight capacity compared to typical color individuals, aligning with the life-history traits of resident morphs [21].

In most cases, intraspecific variation in insects lacks visually discernible traits, yet migrants still exhibit pronounced morphological and physiological differences compared with residents. Altizer and Davis found that the wing morphology of migratory monarch butterflies (*Danaus plexippus*) is significantly larger than that of non-migratory individuals [22]. Likewise, seasonal migratory populations of the cotton bollworm (*Helicoverpa armigera*) exhibit stable differentiation in body size [23,24]. The expression patterns of the circadian rhythm and of juvenile hormone pathway genes in black cutworm (*Agrotis ipsilon*) migrants differ sharply from those in laboratory-reared populations [25]. These findings demonstrate that a cryptic migratory morph can exist even when visual traits are indistinct. Identifying such divergence and developing robust differentiation methods are therefore essential for elucidating the mechanisms of insect migration and for designing accurate monitoring and early-warning systems.

*Spodoptera frugiperda* (fall armyworm, FAW), a pest native to the Americas, has invaded Asia and Africa in recent years, rapidly becoming a major threat to global agricultural production [26,27,28,29]. Since its first invasion into Yunnan, China, in late 2018, it has spread to most regions of the country, posing a serious threat to maize production in China [30,31,32]. Although numerous studies both domestically and internationally have investigated its migration patterns and behavior, whether a distinct migratory ecotype exists remains inconclusive. Chen et al. distinguished migratory individuals from resident individuals based on take-off behavior in the field and found that migrants had slightly longer forewings than residents [33]. Similarly, Sun et al. tethered moths in flight mills and showed that individuals with strong flight capacity exhibited significantly greater body weight, body length, wing length, and wing width compared to those with weak flight capacity [34]. These studies suggest, to some extent, that larger FAW individuals may possess stronger flight capacity. However, it remains unknown whether natural migratory populations of FAW display similar morphological syndromes and whether these traits reflect stable, population-level differentiation.

Yunnan Province has the largest recorded area of FAW occurrence and exhibits the most severe FAW-induced damage in China. Its climate is predominantly tropical and subtropical throughout the year, forming a maize cultivation zone broadly distributed across Yunnan, with peak production areas in the south and northeast [35,36,37]. In southern Yunnan, maize can be planted and harvested two or three times per year, whereas in northeastern Yunnan, it is only planted and harvested once or twice annually, providing abundant food resources and favorable habitats for FAW populations [38]. Each spring and summer, the southwest monsoon carries moths from neighboring countries into southern Yunnan, where they merge with the local year-round breeding population and subsequently migrate eastward and northward into other regions of China [32,39,40]. In autumn, the southerly winds drive FAW populations from surrounding provinces or local areas in Yunnan migrate back into central Yunnan through the north-eastern and eastern corridors, with some even returning to Indo-China Peninsula [3,29,41]. Therefore, southern and northeastern Yunnan serve as critical seasonal migration corridors for the FAW between Southeast Asian countries (Myanmar, Laos, etc.) and China.

To investigate whether FAW migrants exhibit distinct morphological traits and to establish a reliable method for their identification, in this study, we used high-altitude searchlight traps to capture migratory FAWs in southern and north-eastern Yunnan. The morphological parameters of the field migrants and laboratory colony were quantified, and differences in morphology and flight capacity across successive generations were statistically analyzed under laboratory conditions for the migratory populations, aiming to characterize the morphological and biological traits associated with migration. By identifying the migratory ecotype of FAW field populations through appropriate combinations of morphological traits, our results demonstrate the existence of stably differentiated migratory ecotype FAWs in the field. This study provides an important basis for elucidating the migration mechanisms of this pest and for developing regional monitoring and early warning technologies.

## 2. Materials and Methods

### 2.1. Test Population of FAW

Migratory Population: The trapping sites were located at the field migratory pest monitoring stations of the Institute of Plant Protection, Chinese Academy of Agricultural Sciences, in Zhengdong village, Jiangcheng County, Pu’er, Yunnan (101°30′2″ E, 22°31′17″ N) and Gucheng village, Xundian County, Kunming, Yunnan (25°50′34.5″ N, 103°7′12.2″ E). Previous studies have indicated distinct migration peaks of FAW in Pu’er during spring and summer (May–July) and in Kunming during autumn (August–October) each year, with high moth abundance [42,43]. FAWs trapped during these peak periods are considered migratory populations. From April to July and August to October 2023, high-altitude searchlight traps were used at the aforementioned monitoring stations to capture migrating insects (searchlights were operated daily, except during power outages and extreme weather). The searchlights were placed in open areas around the monitoring stations with no obstructions; operation times were determined based on local sunset and sunrise times (Pu’er: https://richurimo.bmcx.com/zhengdongzhen__richurimo/ (accessed on 1 April to 31 July 2023); Kunming: https://richurimo.bmcx.com/jinyuanxiang_he__richurimo/ (accessed on 1 August to 31 October 2023)). Following Feng et al., each searchlight unit comprised a 1000 W metal-halide lamp (JLZ1000BT, Yaming Lighting Co., Shanghai, China) fitted with a GT193 reflector, directing the beam vertically (90°) to ~500 m to attract migratory, positively phototactic insects [44]. A 0.73 m^3^ cage of 120-mesh nylon netting beneath the lamp retained captured moths. FAWs were sorted each morning and immediately stored at −80 °C. Adults collected during the peak periods 9–21 June (Pu’er) and 5–25 September (Kunming) were selected for morphometric analyses.

In addition, approximately 30 pairs of live adults trapped in Pu’er during the same period were sexed (1:1 ratio) and placed in 500 mL plastic cups (one pair per cup) supplied daily with cotton soaked in 10% fresh honey solution. Laid egg masses were used to establish the F1 generation of the migratory strain. F1 larvae were reared on an artificial diet (corn meal, soybean meal, yeast powder, etc.; Liang et al. [45]) until pupation; newly emerged adults (<12 h) were paired as above. Rearing conditions were 25 ± 1 °C, 60 ± 10% RH, L:D = 16:8 h. The migrants trapped using the high-altitude searchlight were maintained for three successive generations (F1–F3) for subsequent morphometrics and tethered-flight tests.

Field population: From September to October 2025, adult FAWs were captured in the field using sex pheromone traps (following the method of He et al. [43]) in Gucheng village, Xundian County, Kunming, Yunnan (25°50′34.5″ N, 103°7′12.2″ E). Trapping was conducted in a flat wheat field (~2000 m^2^) at the 5–7-leaf growth stage, with row spacing ≈ 15 cm and plant spacing ≈10 cm. Four bucket traps (Bioglobal Agricultural Science Co., Ltd., Shenzhen, China) baited with synthetic sex pheromone (main components: Z9-14: Ac and Z7-12: Ac; 12 mg per PVC capillary lure) were arranged in a square (≈50 m side length) at equal distances in the center of the plot. Traps were installed ~1 m above ground; pheromone lures were replaced every 30 days. Each morning, captured moths were collected and immediately stored at −80 °C. Adults trapped during the peak period (20–30 September) were selected for subsequent morphometric measurements.

Laboratory population: In late April 2023, 4th to 6th instar larvae of FAW were collected from fresh corn fields in Zhengdong village, Jiangcheng County, Pu’er, Yunnan (101°30′2″ E, 22°31′17″ N). The larvae were reared under laboratory conditions on fresh corn leaves until pupation. The same protocol used for the migratory strain was followed for adult maintenance and subsequent generations. After seven generations of continuous inbreeding, the stable colony was used for morphometric measurements and tethered-flight assays. To prevent cross-contamination, the laboratory and migratory populations were maintained in separate rooms.

### 2.2. Measurement of Adult Morphological Parameters and Flight Capacity

#### 2.2.1. Measurement of Morphological Parameters

Morphological parameters of moths were quantified following the method described by Yu et al. (Figure S1A) [46]. The body weight of intact moths was measured using a precision balance with 0.1 mg resolution (CP224C, Ohaus International Trading Co., Ltd., Shanghai, China). Wings were then carefully excised, and the maximum length and width of the forewing (recorded as forewing length and width), as well as the forewing area, were measured under a calibrated ultra-depth 3D microscope (VHX-2000, Shanghai Keyence Co., Ltd., Shanghai, China). The maximum length and width of the insect body were measured using an electronic vernier caliper (7D-02150, Forgestar Co., Ltd., Shanghai, China). To account for potential effects of prolonged storage on morphological traits, all migrant and field moths were measured within 24 h of freezing. For laboratory-colony and F1–F3 generations of the migratory population, 2-day-old adults (stored frozen for less than 24 h) were selected for the measurements described above. Morphological parameters were recorded from 472 moths of the migratory strain in total: 138 (51 females, 87 males) from Pu’er and 334 (63 females, 271 males) from Kunming. The laboratory colony was represented by 300 adults (136 females, 164 males) and the field population comprised 104 males, as pheromone traps attract only males. Given that the trends in morphological differences between the migratory and laboratory populations were relatively consistent across sexes (Figure S1B–G’), only females were measured in the F1–F3 generation (n > 100 in each generation).

#### 2.2.2. Flight Performance Assessment

Existing studies indicate that under field conditions, FAWs normally begin migration two days after emergence and may fly on successive nights [29,33,43]. Following Sun et al., intact, healthy 2-day-old females from different populations (laboratory population and F1–F3 generations of the migratory strain) were tethered on 24-channel flight mills (FXMD-24-USB, Jiaduo Science, Industry and Trade Co., Ltd., Hebi, China) and flown either for a single night or three consecutive nights [34]. Each moth was chilled on ice for 5 min, its dorsal thoracic scales were gently brushed off, and a 3-mm plastic ring was glued (cyanoacrylate glue, 20280, Aibida Adhesive Co., Ltd., Guangzhou, China) to the dorsal thorax. Only individuals that fully extended their wings and exhibited active movement were used. Flight tests ran from 20:00 to 08:00 the next morning and were performed at 25 ± 1 °C and 60 ± 10% RH under total darkness. For the three consecutive nights of flight treatment, moths were removed at 08:00, fed 10% honey solution and held under the adult rearing protocol described earlier. Then, they were reattached to the mill at 20:00 for the next flight until it was completed. Raw data were logged automatically; females that accumulated >1 h flight per night were scored as valid. At least 15 valid individuals were obtained for each flight treatment of different strains.

### 2.3. A Method for Distinguishing Migratory-Ecotype Individuals Based on Flight Potential

Given the high correlation between wing area and wing length/width [47], we reduced the number of parameters to retain only five directly measured traits (body mass, length, and width and forewing length and width) for model construction. Raw values were randomly split into training and test sets (8:2); the former was used to construct the model and the latter to validate its effectiveness. No missing values were present in the final training sets; therefore, no imputation was performed. Two derived parameters—WL (Scaled Mass Index (SMI)-corrected) and FA [47,48,49]—were subjected to the same modeling procedure.

WL reflects the mass borne per unit wing area and has been reported to be positively correlated with the minimum sustainable flight speed and energy expenditure, making it a core dimensional index for comparing flight capability. Typically, insects with higher WL require a higher wingbeat frequency or greater energy cost to maintain flight, whereas a lower WL facilitates efficient long-distance flight [50,51]. The FA reflects insect flight fitness, with higher FA favoring better energy conservation during flight [49,52].

First, body mass was corrected with the SMI, following Peig and Green, to remove bias caused by body size variation [53]:

(1) $${SMI}_{i}=M_{i}{\left(\frac{L_{0}}{BL_{i}}\right)}^{b_{SMA}},$$

where *M_i_* is individual body mass, *BL_i_* is body length, *b_SMA_* is the SMA slope of logM and logL fitted to the training set, and *L*_0_ is the geometric mean of body length in the training set.

WL_i_ was then calculated using the following formula according to Chai and Dudley [54]:

(2) $${WL}_{i}=\frac{{SMI}_{i}}{2FS_{i}},$$

where *SMI_i_* serves as the effective mass, and the denominator approximates the combined area of both forewings, with *FS_i_* representing the area of a single forewing.

The FA_i_ was calculated with the formula below, following Flockhart et al. [50]:

(3) $$FA_{i}=\frac{FL_{i}}{FW_{i}},$$

where *FL_i_* is individual forewing length and *FW_i_* is forewing width.

WL and FA were log-transformed in the training set to reduce skewness and scale effects of the odds ratio variables. All traits were then standardized (Z-score, mean = 0, SD = 1) and the same transformations were applied to the test set. The transformed features were input into an elastic-net logistic regression. Within the range of α ∈ {0.2, 0.5, 0.8}, five-fold stratified cross-validation was employed to select α and λ, with the area under the curve value (AUC) used as the criterion for model fitness evaluation. Model-coefficient 95% confidence intervals were obtained from 1000 bootstrap replicates. Because the features had been standardized, the odds ratios (ORs) represent the change in the odds of belonging to the laboratory population relative to the migratory population per one-standard-deviation increase in each feature.

A rigorous probability threshold strategy was employed to calculate the probability of all members of the laboratory population in the training set being predicted as the migratory ecotype, and the 99th percentile of these probabilities was set as the decision boundary. Only if an unknown individual’s predicted probability of being the migratory ecotype was equal or exceeded this threshold was it classified as migratory.

### 2.4. Statistical Analysis

Data were analyzed using SPSS 19 (IBM Corporation, Chicago, IL, USA), and the results are presented as the mean ± SEM (Standard Error of the Mean). Prior to analysis, the normality of the data and homogeneity of variances were verified using the Shapiro–Wilk test and Levene’s test, respectively. Data were log-transformed only when they failed both normality and homogeneity of variance. One-way ANOVA was used to compare (i) morphological traits between the migratory and laboratory populations for each sex, (ii) morphological traits of females between the F1–F3 generations of the migratory population and the laboratory colony, and (iii) the flight performance of females across the groups described in (ii) with different flight treatments. Tukey’s honestly significant difference (HSD) post hoc test was conducted for multiple comparisons when significant differences were detected (*p* < 0.05). All results were visualized using GraphPad Prism 8.0 (GraphPad Software, La Jolla, CA, USA).

## 3. Results

### 3.1. Morphological Differences in FAWs Between Field Migratory Populations and Laboratory Populations

**Figure 1:**
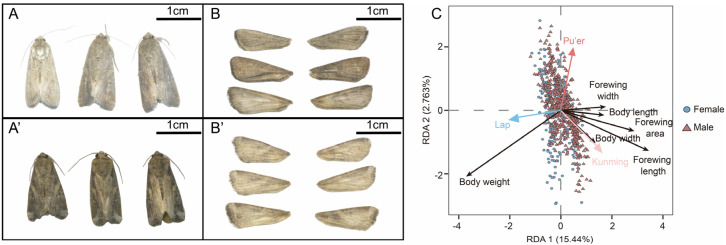
Morphological differences between Lap, Pu’er and Kunming. (**A**,**A’**) Representative photographs of female and male FAWs from Lap, Pu’er, and Kunming populations, respectively (from left to right). Scale bar = 1 cm. (**B**,**B’**) Representative forewing images of female and male FAW from Lap, Pu’er, and Kunming populations, respectively (from top to bottom). Scale bar = 1 cm. (**C**) Redundancy Analysis (RDA). A smaller angle and a longer vector indicate a stronger correlation with the respective population. Lap: laboratory population, Pu’er: migrants from Pu’er, Kunming: migrants from Kunming.

Through quantifying multidimensional morphological parameters (body size, body mass, wing length, etc.), we systematically compared the morphological differences between migratory and non-migratory FAWs (laboratory population). The results revealed that although the body weight of the laboratory population was significantly higher than that of the migratory population (Figure S1B,B’), the migratory moths exhibited significantly greater body lengths (Figure S1C,C’), body widths (except for males from Pu’er) (Figure S1D,D’), forewing lengths (Figure S1E,E’), forewing widths (Figure S1F,F’), and forewing areas (Figure S1G,G’) compared to the laboratory population. Notably, migratory moths from Kunming exhibited a larger body size than those from Pu’er, even though some parameters showed no significant differences (Figure 1A–B’).

Redundancy analysis revealed a significant difference in morphological traits among the three groups (*F*_2,769_ = 85.58, *p* = 0.001). The model explained a total of 18.20% (RDA 1: 15.44%, RDA 2: 2.76%) of the morphometric variation. Migratory moths were primarily distributed in the positive region of the RDA 1 axis, exhibiting greater forewing length, width, and area, as well as body length and body width. In contrast, laboratory-reared individuals were mostly concentrated in the negative region, characterized by a smaller body size but greater body weight (Figure 1C). Moreover, the Pu’er migratory population displayed greater morphological variability than the Kunming population.

Taken together, these results demonstrate that migratory FAW populations possess a larger body size (body length and width) and wing morphology compared to non-migratory populations.

### 3.2. Changes in the Morphological Traits and Flight Capacity of the F1–F3 Generations of Migratory FAW Populations Under Indoor Successive Rearing

To investigate whether the morphological differences among different populations can be stably inherited, we continuously self-crossed the migratory population under laboratory conditions for three generations and recorded the corresponding morphological traits in females of each generation.

**Figure 2:**
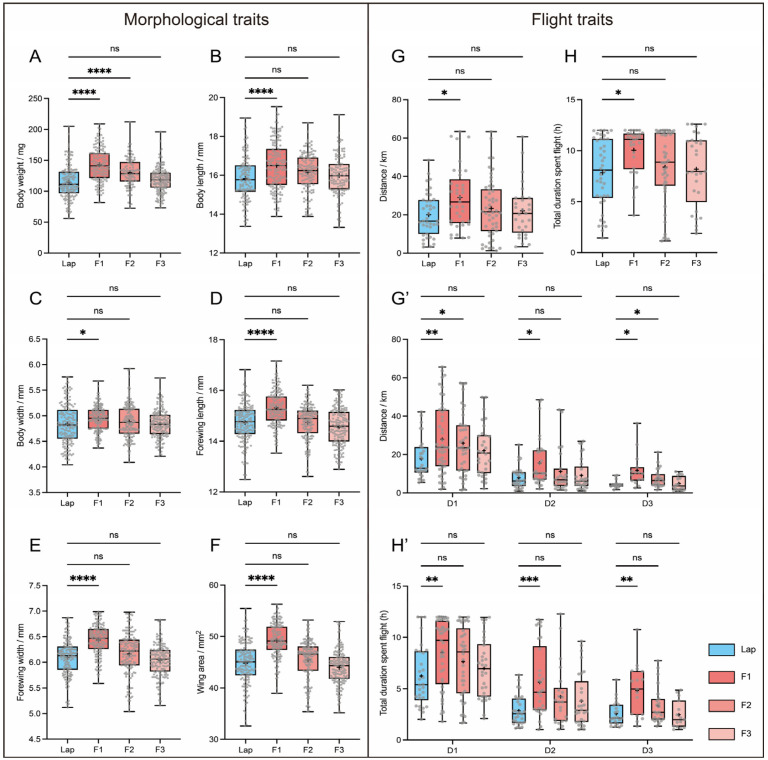
Changes in morphology and flight traits in the migratory population under indoor conditions across F1–F3 generations. (**A**–**F**) Generational changes in body weight, length, and width and forewing length, width and area, respectively, in F1–F3 generations. n > 100 per group. (**G**,**H**) Generational changes in flight distance and duration after one-night tethered flight, respectively, in F1–F3 generations. n > 20 per group. (**G’**,**H’**) Generational changes in flight distance and duration after three consecutive nights of tethered flight, respectively, in F1–F3 generations. n > 15 per group. Lap: Laboratory population. F1, F2, and F3: the first, second, and third generation of the migratory population, respectively. D1, D2 and D3: the first, second, and third night of three consecutive nights of tethered flight. ns, not significant; *, *p* < 0.05; **, *p* < 0.01; ***, *p* < 0.001; ****, *p* < 0.0001.

One-way ANOVA revealed that F1-generation females exhibited significantly greater values in every examined trait than laboratory females: body mass (F1: 143.0 ± 2.30 mg vs. Lab: 114.8 ± 2.19 mg, *p* < 0.0001; Figure 2A), body length (16.50 ± 0.10 mm vs. 15.83 ± 0.10 mm, *p* < 0.0001; Figure 2B), body width (4.94 ± 0.02 mm vs. 4.84 ± 0.03 mm, *p* < 0.05; Figure 2C), forewing length (15.31 ± 0.06 mm vs. 14.74 ± 0.06 mm, *p* < 0.0001; Figure 2D), forewing width (6.43 ± 0.02 mm vs. 6.09 ± 0.03 mm, *p* < 0.0001; Figure 2E) and forewing area (49.25 ± 0.26 mm^2^ vs. 44.77 ± 0.34 mm^2^, *p* < 0.0001; Figure 2F). In the F2 generation, only body mass remained significantly higher than that of Lap (130.0 ± 2.40 mg, *p* < 0.0001); the remaining parameters exhibited no significant differences. By the F3 generation, all morphological parameters were indistinguishable from those of the laboratory population. Thus, our results indicate that the distinctive body-size features of the migratory population are induced by environmental rather than genotypic divergence.

Flight mills were used to quantify one-night and three-consecutive-night flight parameters of 2-day-old females from each migratory generation (with the laboratory population as the control) to investigate changes in flight capacity across generations. After a single 12-h tethered flight in one night, F1 females flew significantly farther (F1: 28.73 ± 3.04 km, Lap: 20.56 ± 2.43 km, *p* < 0.05) (Figure 2G) and longer (F1: 10.03 ± 0.43 h, Lap: 7.16 ± 0.66 h, *p* < 0.001) (Figure 2H) than laboratory females, whereas neither the maximum nor average flight speeds differed between the two groups (Figure S2A,B). The same pattern was observed when moths were flown for three consecutive nights: F1 females again outperformed laboratory females in total flight distance and duration (Figure 2G’,H’), but not in speed (neither maximum speed nor average speed) (Figure S2A’,B’).

All flight parameters in F2—except for the total distance on the first and third nights of continuous flight (F2, 1D: 25.95 ± 2.60 km, Lab, 1D: 17.70 ± 1.94 km, *p* < 0.05; F2, 3D: 7.55 ± 0.97 km, Lab, 3D: 4.57 ± 0.53 km, *p* < 0.05) (Figure 2G’) and the average speed on the second night of continuous flight (F2, 2D: 2.63 ± 0.17 km/h, Lab, 2D: 2.08 ± 0.12 km/h, *p* < 0.05) (Figure S2B’), which were significantly greater in F2 females than in those of the Lap—and all flight parameters in F3 showed no significant differences compared to the laboratory population—mirroring the same generational trend observed in morphological traits.

Collectively, these results indicate that the morphological and flight performance of the migratory population progressively converge toward the non-migratory phenotype under laboratory conditions, suggesting the existence of migratory ecotype differentiation in FAWs.

### 3.3. Identification Methods for the Migratory Ecotype of FAW and Their Utilization in Field Population Discrimination

**Figure 3:**
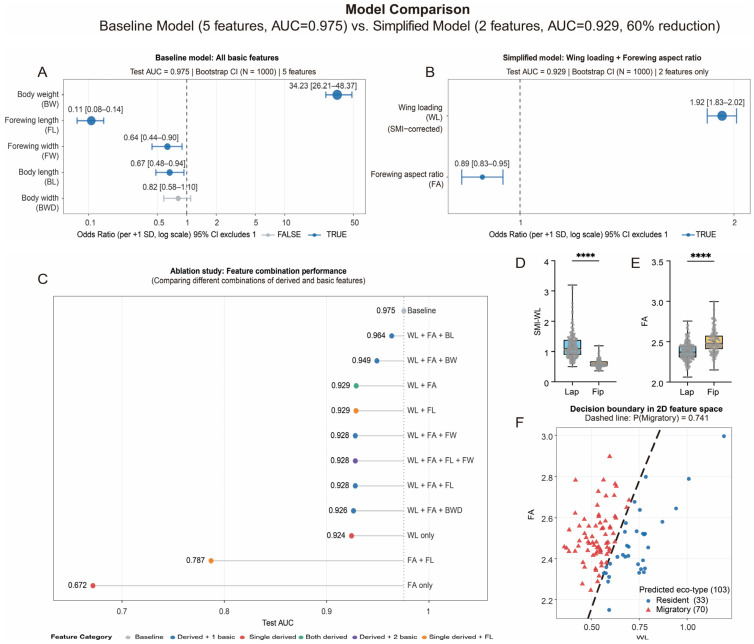
Optimal discriminative indices for the migratory ecotype of FAW predicted by elastic-net logistic regression and its proportions in the field. (**A**,**B**) Performance of the baseline model and the simplified model built from different morphometric parameters. Baseline model: logistic regression using five direct traits (body mass [BW], body length [BL], body width [BWD], forewing length [FL], forewing width [FW]). Simplified model: logistic regression using two derived indices (wing loading [WL], forewing aspect [FA]). Points in the plots represent the median of standardized odds ratios (ORs). The horizontal axis shows the standardized ORs (logarithmic scale); horizontal error bars are 95% bootstrap confidence intervals (n_Bootstrap_ = 1000). (**C**) Ablation analysis: AUC performance of various morphological parameter combinations on the test set. Colors represent different combinations of types. (**D**,**E**) WL and FA for different populations, respectively. n > 100 per group. (**F**) Prediction of migratory ecotypes in Fip. n = 103. Lap: Laboratory population, Fip: field population. ****, *p* < 0.0001.

Five direct morphological traits (body weight, length, and width and forewing length and width) were used to build a baseline model with elastic net logistic regression (test set AUC = 0.975) (Figure S3). The model demonstrated that body weight and length and forewing length and width were the most informative for discriminating the two ecotypes (Figure 3A).

Based on previous research [48,49], we derived two traits—“WL” (SMI-corrected) and “FA”—and modeling was conducted, taking care to strictly avoid information leakage. This simplified model achieved an AUC = 0.929 and met the standard on the test set (Figure 3B and Figure S3). Collinearity assessment revealed that for the simplified model, r = −0.14 and VIF = 1.032 (Table S1). Consistent with previous studies, the model results indicate that individuals with higher WL are more likely to be classified as the resident (non-migratory) ecotype, while individuals with a larger FA are more likely to belong to the migratory ecotype [48,49,50,51].

Different combinations of the two derived traits and five baseline traits were evaluated to further screen for suitable indicators. The ablation model results show that across all subsets, AUC varied only marginally. The baseline model using the five direct morphological traits yielded the highest AUC value, whereas the simplified model with two derived indices achieved near-equivalent performance (AUC = 0.929) with 60% fewer predictors and effectively eliminated multicollinearity among the five direct traits (Figure 3C). Based on performance, interpretability, and reproducibility, we selected the combination of WL and FA as the optimal morphological parameter set for identifying migratory ecotypes.

Morphometric profiling of the Fip revealed that WL was significantly lower than that in the Lap (Lap: 1.19 ± 0.03 vs. Fip: 0.60 ± 0.01, *p* < 0.0001; Figure 3D), whereas FA was markedly higher (Lap: 2.39 ± 0.01 vs. Fip: 2.50 ± 0.01, *p* < 0.0001; Figure 3E). Using the simplified model’s two-dimensional decision boundary (probability threshold of 74.1%) to classify the field populations, approximately 70% of the individuals were identified as the migratory ecotype (Figure 3F). Consistent with the findings of He et al., our results confirm that the majority of individuals captured by pheromone traps in the field are of the migratory population [43] and demonstrate that combining WL and FA provides a robust, field-ready discriminator for the migratory ecotype of FAW.

## 4. Discussion

Morphological differentiation within a single species is widespread among migratory insects and has long provided an intuitive means of distinguishing migrants from residents [16,55,56]. In this study, by comparing multiple morphological parameters between the migratory population and the laboratory population of FAW, we found that migrants exhibited lower body mass yet longer body length, wider body width and larger wings. These morphological traits, together with flight capacity, converged to laboratory levels within three indoor generations, indicating that a stably differentiated migratory ecotype persists in FAW. Using WL and FA as discriminators, we further showed that the migratory ecotype constitutes a high proportion of field FAW populations, demonstrating that specific morphological traits can effectively differentiate FAW migrants.

Given its unique topography and monsoon climate, Yunnan serves as a crucial migratory corridor for FAWs entering southwestern China and migrating northward [29,39,43]. Here, we collected wild migrants at several Yunnan sites with high-altitude searchlight traps and compared their morphometrics with those of a laboratory-reared population. RDA revealed the multidimensional correlations between morphology and migratory behavior: the RDA model explained a total of 17.98% of the morphological variation (RDA 1: 14.9%, RDA 2: 3.071%), with migrants exhibiting larger body dimensions (length, width) and bigger wings, corroborating the validity of using multidimensional morphological traits to discriminate the migratory ecotype. Intriguingly, laboratory moths were significantly heavier than migrants. We hypothesize that laboratory-reared individuals can replenish nutrients ad libitum with honey solution, whereas field moths rely solely on dilute nectar or dew, making it difficult to offset the energetic cost of long-distance flight [57]. In addition, a lower body mass coupled with larger wing area yields reduced wing loading—an aerodynamic prerequisite for efficient flight [52,58,59,60]. It should be noted that, although the morphological divergence between migrants and the laboratory population is clear, broader field surveys are needed to confirm the stability of these traits across time and space.

Notably, our results revealed that migratory moths in Kunming exhibited significantly larger morphometric parameters compared to those from Pu’er. Similarly, Zhou et al. observed pronounced seasonal variation in the body size of *H. armigera* migrating through the Bohai Bay region, with southward-moving populations displaying lower morphological traits than their northward counterparts [24]. Given the diversity of host plants at different FAW source locations and the variation in crop phenology across regions, we hypothesize that nutritional differences during the larval stage—stemming from disparate food sources—constitute a key factor driving the observed morphological divergence among geographically distinct migratory ecotypes of FAW [61,62].

Numerous studies have demonstrated that environmental variables (e.g., temperature, humidity, photoperiod), population density, and host-plant nutrition are key regulators of intraspecific variation in migratory insects [13,16,19,63]. Here, we self-crossed the migratory ecotype for three generations under stable laboratory conditions and tracked their morphometrics. The F1 generation was significantly larger than the resident colony in every trait, yet most migratory-specific differences disappeared unexpectedly in F2 and had completely vanished by F3, at which point all indices matched the laboratory strain. This indicates that environmental cues—rather than dietary differences—are the primary drivers in switching FAWs from the migratory to the resident ecotype. Intriguingly, field migrants were markedly lighter than residents, whereas the F1 generation was heavier than residents. Taken together with the RDA results, we hypothesize that the F1 generation reared under favorable conditions represents a critical transitional period at which the migratory ecotype begins to shift toward the resident form. Nevertheless, which specific environmental factors trigger this switch, at which larval instar exposure to such environments leads to adult transition, and whether the resident ecotype can revert to the migratory ecotype under certain stresses, warrant further investigation.

Insect body size is, to some extent, associated with flight capability. Monarch butterfly residents are noticeably smaller than migrants [22], while northward-migrating cotton bollworms (which exhibit longer flight durations) are longer and wider than southward-migrating populations [24]. In FAWs, field populations with higher take-off rates possess larger forewings, and individuals demonstrating strong tethered flight capacity also exhibit a larger body size [33,34]. Taken together, these findings hint at a positive relationship between body size and flight ability. Consistently, we found that F1 females flew significantly farther and longer than laboratory moths in both single and multi-night tests, while F2 moths exceeded the laboratory strain only in a few flight metrics, and by F3, no differences remained. This parallel decline in both morphology and flight capacity indicates that, under laboratory conditions, the characteristics of the migratory ecotype FAW progressively revert toward those of the resident form over successive generations. However, in this study, only females were examined, and whether males exhibit the same transitional trend requires further investigation.

Even during peak migration periods, a considerable number of resident individuals remain in the field, leading to a high rate of mixed captures, especially when using bio-attractant traps. Traditional methods that discriminate migrants by examining ovarian or testicular development are time-consuming, destructive, and preclude subsequent behavioral assays [29,43,64]. In this study, we propose the use of elastic-net logistic regression on an array of morphometric traits as a non-lethal, high-accuracy approach that relies on WL (SMI-corrected) and the FA to efficiently identify the migratory ecotype of FAW. Applying the decision boundary derived from these two traits, we estimated that approximately 70% of the Kunming field population belongs to the migratory ecotype, a result that is highly consistent with the findings of He et al. [43]. This study provides a novel, efficient tool for rapidly and reliably distinguishing migratory ecotypes in FAW.

However, this study focused solely on morphometrics and flight capacity; whether other key physiological and biochemical traits, such as fecundity, energy metabolic efficiency, and detoxification capacity, also exhibit significant differentiation between the two ecotypes requires further in-depth investigation and mechanistic elucidation. Considering that the Yunnan region serves as a profound seasonal migration corridor for FAWs, it is necessary to integrate modern information technologies to accelerate the development of automated, high-throughput insect morphometric devices in the future. Coupled with real-time monitoring and our methods of distinguishing migratory-ecotype FAWs, comprehensive control measures—including insect-attracting light traps, biological attractants, and genetically modified crops—should be deployed in a timely manner to reduce the population base of migrating FAWs and thereby mitigate the risk of cross-regional outbreaks of FAWs at the source [65].

In summary, this study confirms the existence of migratory ecotypes in FAW and demonstrates that the morphology and flight performance of migrants revert toward resident levels within a few generations under favorable conditions. We also provide an efficient, field-ready strategy to discriminate the migratory ecotype and demonstrate that field FAWs captured by pheromone traps are mainly migrants. These findings deepen our understanding of intraspecific phenotypic plasticity in insects and open new avenues for pest monitoring, early warning and area-wide management.

## Data Availability

The original contributions presented in this study are included in the article/Supplementary Material. Further inquiries can be directed to the corresponding author.

## Supplementary Materials

The following supporting information can be downloaded at https://www.mdpi.com/article/10.3390/insects17010095/s1. Figure S1: Differences of multiple morphological traits between Lap, Pu’er and Kunming. (A) Schematic of length- and width-related morphological measurements in FAW. (B–G) Female body weight, length, and width and forewing length, width, and area, respectively, across different populations. n > 50 per group. (B’–G’) Male body weight, length, and width and forewing length, width, and area, respectively. n > 80 per group. Lap: laboratory population, Pu’er: migrants from Pu’er, Kunming: migrants from Kunming. Different lowercase letters indicate significant differences (*p* < 0.05); Figure S2: Changes in average and maximum flight speeds in the migratory population under indoor conditions across F1–F3 generations. (A,B) Generational changes in maximum and average flight speeds after one-night tethered flight, respectively, in F1–F3 generations. n > 20 per group. (A’,B’) Generational changes in maximum and average flight speeds after three consecutive nights of tethered flight, respectively, in F1–F3 generations. n > 15 per group. Lap: Laboratory population. F1, F2, and F3: the first, second, and third generation of the migratory population, respectively. D1, D2 and D3: the first, second, and third night of three consecutive nights of tethered flight. ns, not significant; *, *p* < 0.05. Figure S3: ROC curve comparison of the two models; Table S1: Comparison of the VIF value between the two models.
