# Supplementary material for: Morphological Trait Analysis Showed the Existence of a Migratory Ecotype in the Fall Armyworm, Spodoptera frugiperda
Source: Insects. 2026 Jan 14;17(1):95. doi: 10.3390/insects17010095 (PMC12842096; doi:10.3390/insects17010095)
Supplement: Supplementary file 1 [file insects-17-00095-s001.zip › Table S1.pdf]

Table S1:=Comparison of Variance Inflation Factors (VIF) between Baseline and Simplified Models

**Feature**	**Baseline** *(5 features)*	**Simplified** *(2 features)*
Body weight	5.116	NA
Body length	1.914	NA
Body width	1.955	NA
Forewing length	4.344	NA
Forewing width	1.956	NA
Wing loading (SMI)	NA	1.032
Forewing aspect ratio (L/W)	NA	1.032
**Max VIF**	**5.116**	**1.032**
**Mean VIF**	**3.057**	**1.032**
N features	5	2

Note. VIF = Variance Inflation Factor; NA = Not Applicable. The Simplified model was selected as the final model due to significantly lower multicollinearity (Max VIF: $1.032 < 5.116$).
