# Supplementary figures and images for: Morphological Trait Analysis Showed the Existence of a Migratory Ecotype in the Fall Armyworm, Spodoptera frugiperda
Source: Insects. 2026 Jan 14;17(1):95. doi: 10.3390/insects17010095 (PMC12842096; doi:10.3390/insects17010095)

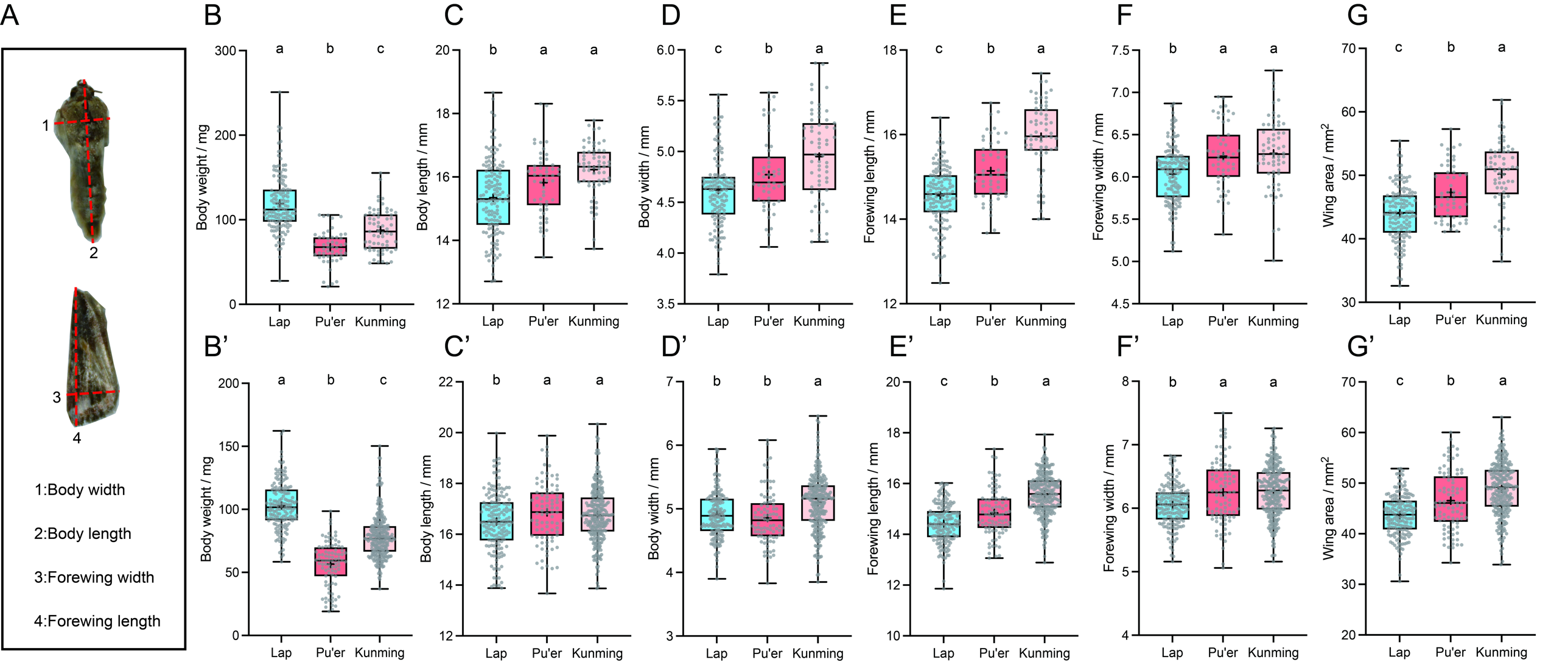

Supplement: Supplementary file 1 [file insects-17-00095-s001.zip › Figure S1.jpg]

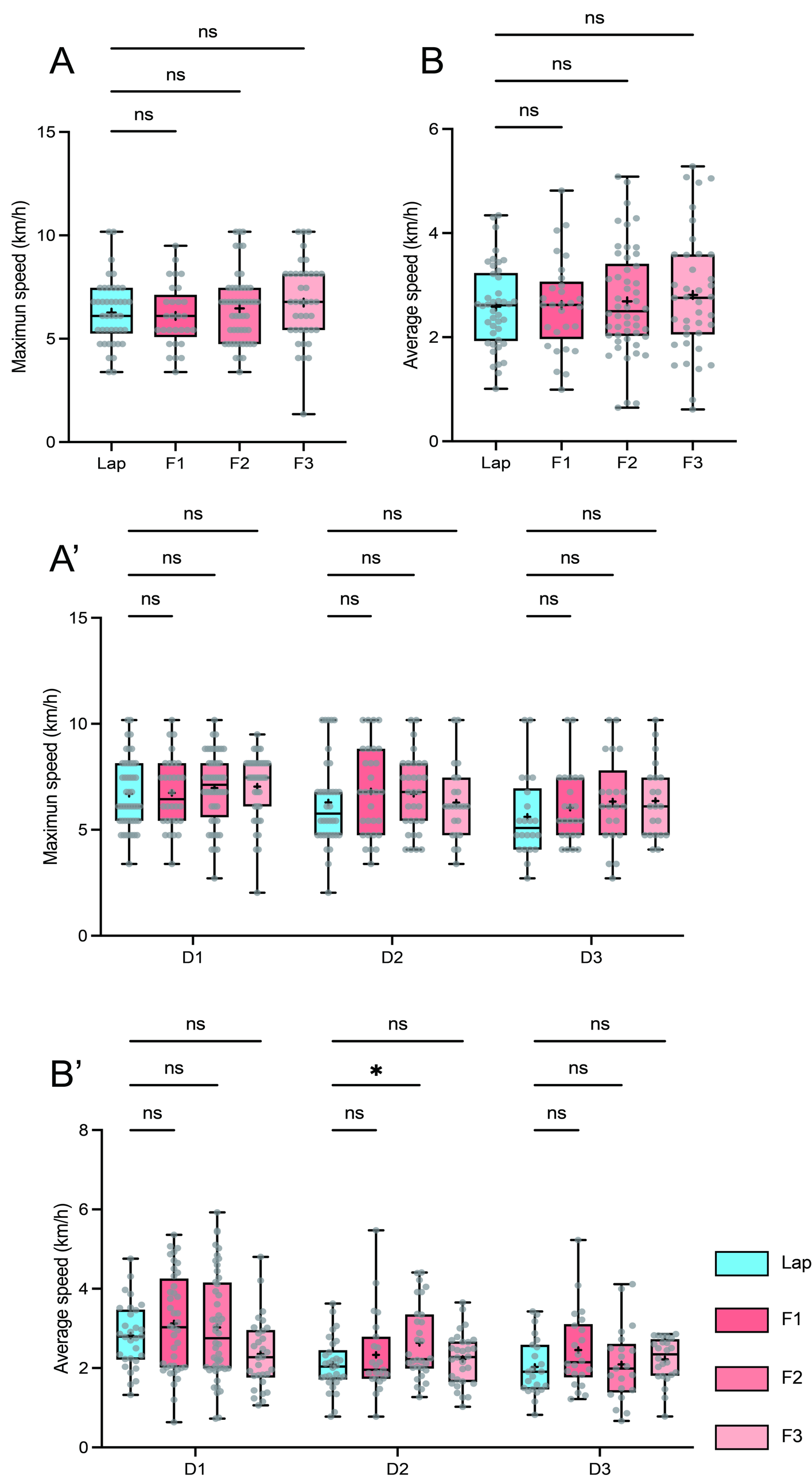

Supplement: Supplementary file 1 [file insects-17-00095-s001.zip › Figure S2.jpg]

# ROC Curve Comparison

Baseline vs. Simplified Model

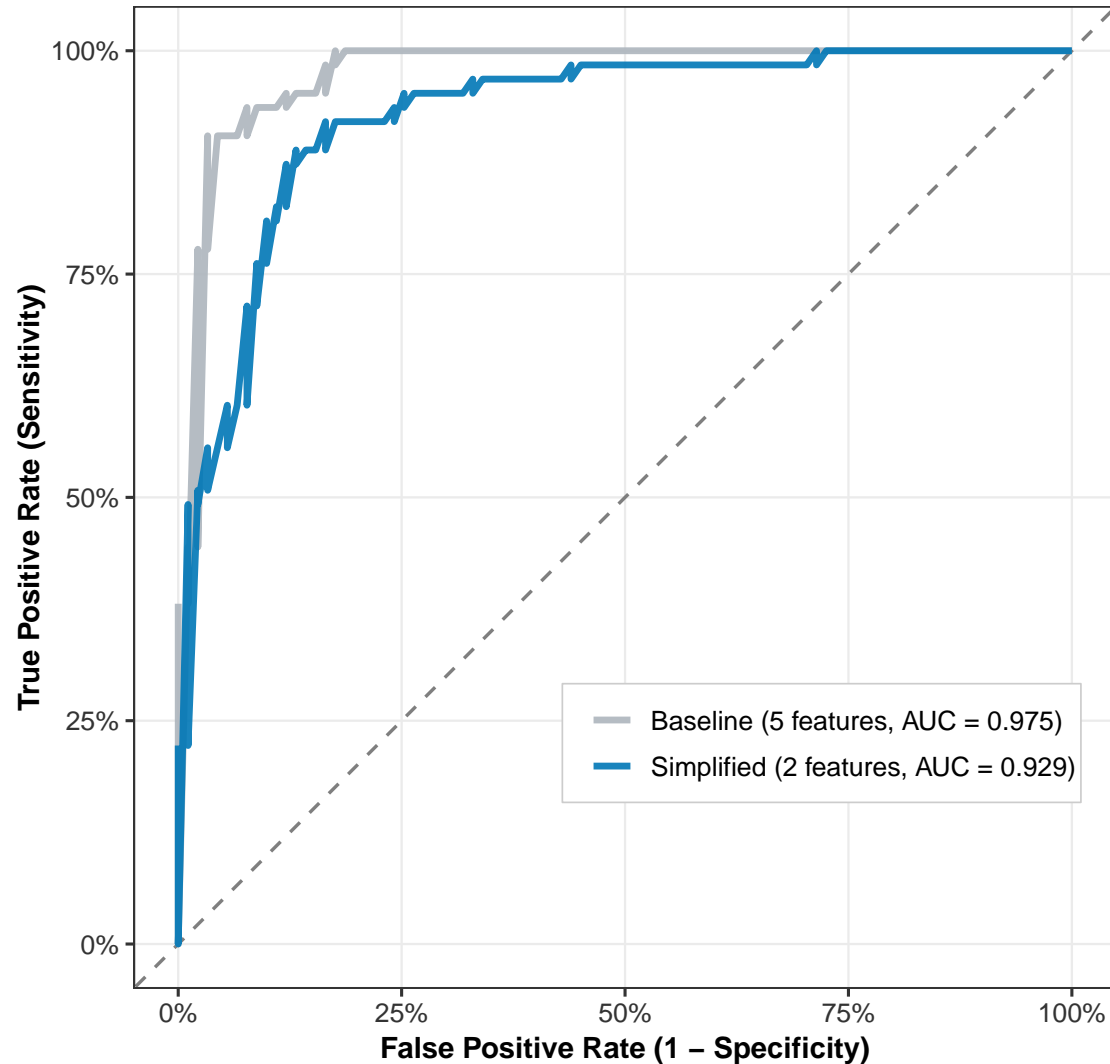

Supplement: Supplementary file 1 [file insects-17-00095-s001.zip › Figure S3.pdf]
